# Left ventricular diastolic function assessed with cardiovascular magnetic resonance imaging and exercise capacity in patients with non-obstructive hypertrophic cardiomyopathy

**DOI:** 10.1186/1532-429X-11-S1-P238

**Published:** 2009-01-28

**Authors:** Lukasz A Malek, Jolanta Misko, Mariusz Klopotowski, Lidia Chojnowska, Mateusz Spiewak, Barbara Milosz, Renata Maczynska, Ewa Piotrowicz, Witold Ruzyllo

**Affiliations:** Institute of Cardiology, Warsaw, Poland

**Keywords:** Cardiovascular Magnetic Resonance, Exercise Capacity, Leave Ventricular Mass Index, Left Ventricular Diastolic Function, Stroke Volume Index

## Introduction

In patients with non-obstructive hypertrophic cardiomyopathy (HCM) and preserved left ventricular (LV) systolic function, diastolic dysfunction is one of the major factors contributing to limited exercise capacity.

## Purpose

To assess the usefullness of simple cardiovascular magnetic resonance (CMR) parameters of LV diastolic function at rest and exercise capacity in patients with non-obstructive HCM and preserved LV systolic function.

## Methods

The study included 13 patients who underwent cardiopulmonary exercise testing on treadmill and CMR within 1 month distance. Analyzed parameters of diastolic function included: atrio-ventricular plane descent (AVPD), sphericity index (SI), left ventricular mass index (LVMI), peak filling rate normalized to LV stroke volume index (PFR/LVSVI) and time from the end-systole to PFR normalized to heart rhythm (TPFR).

## Results

There was a significant correlation between PFR/LVSVI at rest and peak oxygen uptake (V0_2_peak) (r = 0.64, p = 0.02). Patients with V0_2_peak below median (<30 ml/kg/min) had a significantly lower PFR/LVSVI then patients with higher V0_2_peak [5.12 m^2^/s, interquartile range (IQR) 4.16–6.82 vs. 7.93 m^2^/s, IQR 7.49–8.21 respectively, p = 0.035). AVPD, SI, LVMI, TPFR were not related to exercise capacity. There was also no correlation between V0_2_peak and age (r = -0.38, p = 0.19), LV ejection fraction (r = -0.36, p = 0.22) or normalized LV volume indices: LVEDVI (r = 0.09, p = 0.76), LVESVI (r = 0.34, p = 0.26).

## Conclusion

Assessment of LV diastolic function by peak filling rate normalized to stroke volume index with means of CMR at rest in patients with non-obstructive hypertrophic cardiomyopathy and preserved LV systolic function is a usefull predictor of exercise capacity. It can be added to CMR study protocols of HCM patients.

